# DegreeCox – a network-based regularization method for survival analysis

**DOI:** 10.1186/s12859-016-1310-4

**Published:** 2016-12-13

**Authors:** André Veríssimo, Arlindo Limede Oliveira, Marie-France Sagot, Susana Vinga

**Affiliations:** 1 IDMEC, Instituto Superior Técnico, Universidade de Lisboa, Lisboa, 1049-001 Portugal; 2 Instituto Superior Técnico, Universidade de Lisboa, Lisbon, 1049-001 Portugal; 3 Instituto de Engenharia de Sistemas e Computadores: Investigação e Desenvolvimento (INESC-ID), Lisbon, 1000-029 Portugal; 4 ERABLE, Inria, Villeurbanne, France; 5 Laboratoire de Biométrie et Biologie Évolutive, Université de Lyon, CNRS UMR 5558, F-69622, Villeurbanne, France

**Keywords:** Regularization, Cox proportional models, Network metrics

## Abstract

**Background:**

Modeling survival oncological data has become a major challenge as the increase in the amount of molecular information nowadays available means that the number of features greatly exceeds the number of observations. One possible solution to cope with this dimensionality problem is the use of additional constraints in the cost function optimization. Lasso and other sparsity methods have thus already been successfully applied with such idea. Although this leads to more interpretable models, these methods still do not fully profit from the relations between the features, specially when these can be represented through graphs. We propose DegreeCox, a method that applies network-based regularizers to infer Cox proportional hazard models, when the features are genes and the outcome is patient survival. In particular, we propose to use network centrality measures to constrain the model in terms of significant genes.

**Results:**

We applied DegreeCox to three datasets of ovarian cancer carcinoma and tested several centrality measures such as weighted degree, betweenness and closeness centrality. The a priori network information was retrieved from Gene Co-Expression Networks and Gene Functional Maps. When compared with Ridge and Lasso, DegreeCox shows an improvement in the classification of high and low risk patients in a par with Net-Cox. The use of network information is especially relevant with datasets that are not easily separated. In terms of RMSE and C-index, DegreeCox gives results that are similar to those of the best performing methods, in a few cases slightly better.

**Conclusions:**

Network-based regularization seems a promising framework to deal with the dimensionality problem. The centrality metrics proposed can be easily expanded to accommodate other topological properties of different biological networks.

**Electronic supplementary material:**

The online version of this article (doi:10.1186/s12859-016-1310-4) contains supplementary material, which is available to authorized users.

## Background

Precision medicine shows the promise of additional efficacy by bringing more information into the diagnosis process. It is, however, highly dependent on rapid advances in science and technology as data analysis and knowledge discovery techniques are indeed struggling to keep pace with the challenges related to what computer scientists have called *big data* [1]. In this regard, dealing with the high-dimensionality of patients’ data represents a largely unsolved problem, especially when the number of features or covariates involved, such as related to molecular data (which can easily reach tens of thousands), greatly outnumbers the observations (typically in the hundreds). This fact severely hampers the modeling task, usually leading to a degradation in the classifier accuracy and a greater difficulty in extracting knowledge from data [2, 3]. Furthermore, datasets suffering from this curse of dimensionality often lead to over-fitted models which, although they represent the training data, exhibit a significant decrease in their accuracy on new observations [4]. This problem may persist even when feature selection and validation schemes are used. One possible solution to tackle this problem is to impose further constraints on the solution space. This can be accomplished through regularization methods, that penalize more complex structures of the solution space. The goal is to penalize the cost function (e.g. quadratic error, log-likelihood) with additional functions in order to impose a structure on the parameter space.

For linear regression, a regularization method that is widely used is LASSO - Least Absolute Shrinkage and Selection Operator [5], which penalizes the error function with the L1 norm of the regression parameters, leading to a sparse solution. Other possible regularizers include feature or group sparsity, smoothness of the features’ coefficients, or a graph representing how the features are connected [5–11].

These techniques have led to models that are partially capable of dealing with the dimensionality problem and, additionally, are able to improve model interpretability [12–14].

In this context, survival analysis in oncology research represents one of the most challenging areas of application, with the recent development of public databases such as *TCGA* - The Cancer Genome Atlas [15]. Survival analysis involves modeling the time to an event of interest, by uncovering relationships between the given covariates and time distributions [16], and allowing for censored observations (for which the event does not occur). The Cox proportional hazard model [16] is used to model these relationships and has been widely applied in this context. However, it also exhibits problems for datasets with more covariates than observations. For example, using genomic data to determine the relationship of the expression levels of thousands of genes to a death event leads to an under-determined problem that can have multiple solutions.

Recent efforts to combine Cox modeling with regularization techniques have already shown promising results [11, 17, 18]. In particular, sparse models have been developed to identify a small set of gene signatures related to high or low risk patients. Furthermore, the predictability of the model was tested with datasets from five geographically distant populations [17]. Cox regularized models have also been used to predict a patient’s risk of conversion from a mild cognitive impairment to Alzheimer’s disease [18].

Besides these sparsity methods, other techniques tried to embed network-based regularizers, following work on group sparsity [19]. When the features can be connected through a graph, one can further explore this structure in order to improve the models. One example is to impose smoothness on the parameters associated with connected features (in the network). This technique provided good results for modeling survival of ovarian cancer patients where the features correspond to gene expression data [14]. Since there is an underlying structure on the gene feature space given by the patterns of co-expression, these correlations can be applied as constraints to the Cox proportional hazards model. Although the results are promising, there are still few studies that fully explore the network properties of the feature space beyond this connectivity.

In this context, we propose and explore a novel network-degree-constraint Cox proportional hazard model, that we called DEGREECOX, which uses a priori knowledge to leverage the correlation or functional information present in gene expression data. In this survival model, a graph degree constraint is introduced that expresses the importance of a gene by how highly connected it is in the overall network.

We applied DEGREECOX to identify gene expression signatures associated with survival of ovarian carcinoma patients. This type of cancer is the fifth-leading cause of cancer death in US women [20]. DEGREECOX was applied to three large-scale ovarian cancer gene expression datasets [20–22] to predict a patient’s risk and to identify genes associated to death events. We compared DEGREECOX with similar methods such as NET-COX [14] and elastic net [6]. Our results show that using vertex degree can improve the model in terms of its generalization capability.

The code to reproduce the results is available at http://sels.tecnico.ulisboa.pt/gitlab/averissimo/degree-cox.

## Methods

The proposed method DEGREECOX is based on applying network-based regularizers in Cox proportional hazards model estimation. This section will overview several regularizers based on centrality measures of a network and will briefly describe which networks can be applied in the context of gene expression data. Survival models and regularization in the context of Cox regression are then overviewed.

### Network centrality metrics

A biological network is represented as a graph *G*:=(*V,E*), with *V* denoting the set of vertices, or nodes, and *E* the set of edges. In the present context of gene networks, *G* represents the co-expression or functional map network where the vertices are *P* genes, with *P*:=|*V*|, and edges represent a weighted relation between two genes. The graph *G* may also be represented by a *P*×*P* positively weighted adjacency matrix that we denote by **W**.

The matrix **W** is further normalized, leading to the matrix **S** with $s_{ij} = w_{ij} \cdot \left (\sum ^{P}_{n=1} w_{in} \right)^{-1/2} \cdot \left (\sum ^{P}_{n=1} w_{nj} \right)^{-1/2}$, i.e., each normalized value in **S** is obtained by dividing the weights by the square root of the sum over all rows and columns.

**Fig. 1:**
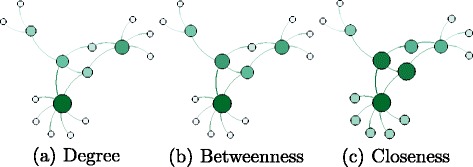
Centrality measures

Network centrality measures characterize each vertex in a network, creating a ranking of the most relevant ones [23]. Research on this topic emerged in the 1950s on the role of central vertices in social networks [24–26]. Different metrics have been proposed in the literature. These typically use network topology to define a function that determines a measure for vertex *y*_*i*_. Among the proposed methods to classify important vertices are degree, betweenness and closeness centrality, briefly described below and illustrated in Fig. 1, where the size and color of a vertex reflect the importance of the vertex for each method.

In the Section, all these measures will be tested on real datasets in order to choose the best ones to be integrated in the proposed regularizer.

#### Degree centrality

The degree of a vertex is the number of its adjacent vertices. Vertices with a high degree are called hubs and may bridge the path between other low degree vertices in the network keeping the network diameter low. The simplest description of network centrality based on the degree of a vertex was first presented by Nieminem [27] and counts the adjacent edges of vertex *y*_*i*_:

1 $$  d_{i} = \sum\limits^{P}_{j=1}a_{ij},  $$

where *a*_*ij*_=1 if vertices *y*_*i*_ and *y*_*j*_ are connected and *a*_*ij*_=0 otherwise.

Extensions of this definition to include weighted networks have been proposed, where the values *s*_*ij*_ represent the normalized weight of the connecting edge instead of a binary value [28, 29]:

2 $$ d_{i} = \sum\limits^{P}_{j=1}s_{ij}.  $$

Methods to determine the centrality of a vertex are local, since they are functions of the neighborhood of *y*_*i*_, therefore not taking into account global properties. For a comparison of multiple networks, this value should be normalized by the total number of vertices [23].

#### Betweenness centrality

The betweenness centrality *B*_*i*_ is equal to the frequency of the presence of vertex *y*_*i*_ in the shortest paths between every two vertices (*y*_*j*_,*y*_*k*_) in the network, *i*≠*j*≠*k*. This will rank vertices by their importance on the communication flow of the network. It may be used to identify possible bottlenecks or relevant regulators of the network. It is defined by:

3 $$  B_{i} = \sum\limits_{j=1}^{P} \sum\limits_{k:k>j}^{P}\frac{g_{jk}(y_{i})}{g_{jk}},  $$

where *g*_*jk*_ is the number of shortest paths between *y*_*j*_ and *y*_*k*_ and *g*_*jk*_(*y*_*i*_) is the number of shortest paths that include vertex *y*_*i*_. Computation of this metric for dense graphs can be done in *Θ*(|*V*|^3^) time and for sparse graphs in *O*(|*V*|^2^· log(|*V*|)+|*V*|·|*E*|) time.

#### Closeness centrality

The idea that the centrality of a vertex is related to its connectivity in the network was suggested by [24, 25]. This measure, denoted by ***C***, is based on calculating, for each vertex *y*_*i*_, its distance *g*_*ji*_ to every vertex *y*_*j*_, *j*≠*i*, in the network, defined as the length of the corresponding shortest path, summing all these distances and taking the inverse:

4 $$ c_{i}^{-1} = \sum\limits_{j \neq i}^{P} g_{ji}.  $$

The rationale is that the more central vertices have lower total distances from all other vertices. This measure requires that the graph is connected, as two disconnected vertices are at an infinite distance from one another.

### Gene networks

In order to apply a network-based regularizer, two types of gene networks will be used: 1) Gene Co-expression Networks (GCN); and 2) Gene Functional Maps (GFM). Both networks consider genes as vertices and the weight of each edge corresponds to the association between the connected genes, which can be the correlation between gene expression or functional annotation.

A gene co-expression network (GCN) is specific for each dataset and is generated using the ranking of Pearson’s correlation coefficients between gene *g*_*i*_ and *g*_*j*_, for all genes in the dataset [30]. The resulting matrix **M**, is given by $M^{-1}_{ij} = r_{ij} \cdot r_{ji}$, where *r*_*ij*_ is the position of gene *g*_*j*_ in the correlation ranking of gene *g*_*i*_.

A gene functional map (GFM) describes the functional activity and corresponds to an interaction network that includes information from ∼30,000 genome-scale experiments and ∼25,000 human genes. It was built using a regularized Bayesian integration system proposed by Huttenhower and colleagues [31] and is available at http://giant.princeton.edu/. Each edge between two genes is probabilistically weighted based on experimental evidence which integrates many different datasets. The functional map used in the present work includes 7562 genes inferred from experiments using ovarian cells.

### Cox proportional hazards model

Given *D*=((***X***_1_,*Y*_1_,*δ*_1_),⋯,(***X***_*n*_,*Y*_*n*_,*δ*_*n*_)), where ***X***_*i*_, *i*=1,…,*n* is the gene expression profile of *n* patients over *P* genes, $\boldsymbol {X}^{\prime }_{i} = (X_{i1},\cdots,X_{iP})$, *Y* is the response variable that indicates the survival time for patient *i* and *δ*_*i*_ is an indicator of whether patient *i* has observed the event (*δ*_*i*_=1) or not (*δ*_*i*_=0). The hazard function for a patient given his expression profile is given by:

5 $$ h(t|\boldsymbol{X}_{i})=h_{0}(t) \exp (\boldsymbol{X}^{\prime}_{i}\boldsymbol{\beta}),  $$

where ***β***=(*β*_1_,⋯,*β*_*P*_) is a vector of regression coefficients and *h*_0_(*t*) is the baseline hazard function. The regression coefficients are estimated by maximizing the Cox’s partial log-likelihood:

6 $$ l(\boldsymbol{\beta}) = \sum\limits_{i=1}^{n} \delta_{i} \left \{ \boldsymbol{X}^{\prime}_{i} \boldsymbol{\beta} - \log \left [\sum\limits_{j:y_{j} \ge y_{i}}^{n} \exp\left (\boldsymbol{X}^{\prime}_{j}\boldsymbol{\beta}\right) \right ] \right \}.  $$

One of the most used estimators for the baseline hazard is the Breslow estimator [32] given by:

7 $$ \hat{h}_{0}(t_{i}) = \frac{1}{\sum_{j:y_{j}\ge t_{i}}^{n} \exp (\boldsymbol{X}'\boldsymbol{\beta})}.  $$

The partial likelihood and the Breslow estimator are induced by the total log-likelihood:

8 $$ \begin{aligned} l({\beta},h_{0}) = \sum\limits^{n}_{i=1} & -\exp(\boldsymbol{X}_{i}'{\beta})H_{0}(t_{i}) + \\ & \delta_{i} \left [ \log(h_{0}(t_{i}))+\boldsymbol{X}_{i}'{\beta} \right ], \end{aligned}  $$

with

9 $$ H_{0}(t_{i})=\sum_{t_{k}\le t_{i}}h_{0}(t_{k}).  $$

The inference of the optimal coefficients ${\hat {\beta }}$ is done by maximizing the total log-likelihood in two steps, alternating between maximizing with respect to ***β*** and updating the *h*_0_(*t*) estimation (in Eq. 7).

### Regularized Cox regression

When the number of gene features *P* is much larger than the observations *n* (*n*≪*P*), the estimation procedure exhibits identifiability problems. In fact, applying the standard Cox proportional hazard model to infer parameters will lead to multiple possible solutions with a large number of non-zero parameters, which severely hampers the classification of new observations.

#### LASSO and RIDGE regression

Strategies that can be used to minimize this problem include the application of *L*_1_ and *L*_2_ norms, in order to restrict the solution space, in particular imposing sparsity and small coefficients for the parameters [5, 6, 33]. This can be done by penalizing the total log-likelihood with a weighted sum of the *L*1 and *L*2 norms, a method called elastic net [6]:

10 $$ \begin{aligned} l_{L_{1}L_{2}}({\beta},h_{0}) = & \sum\limits^{n}_{i=1} \left\{ -\exp(\boldsymbol{X}_{i}'{\beta})H_{0}(t_{i}) + \right. \\ & \left. \qquad \delta_{i}\ \left [ \log(h_{0}(t_{i}))+\boldsymbol{X}_{i}'{\beta} \right ] \right\} \\ & - \frac{1}{2}\lambda\left (\alpha |{\beta}|_{1} + (1-\alpha)|{\beta}|^{2}_{2} \right), \end{aligned}  $$

where *λ* is the parameter controlling the penalizing weight and *α* the balance between the two norms. In particular, *α*=0 leads to the RIDGE regression and when *α*=1, LASSO regression is obtained.

The R package “glmnet” [11] was used to estimate the coefficients with this type of regularizer.

#### NET-COX regression

In the NET-COX model previously proposed [14], a Laplacian matrix constraint is introduced as a smoothness operator among adjacent coefficients in the network. This operator adds a cost, for every pair of genes connected by an edge, which is proportional to the edge weight and the difference between their coefficients. This hypothesis determines that genes that are connected should be correlated. This implies that the coefficients of the features related to the genes should be similar, i.e., vary smoothly through the network.

The Laplacian is then given by:

11 $$ \begin{aligned} \Psi({\beta}) & = \frac{1}{2}\sum\limits_{i,j=1}^{p}S_{ij}\left(\beta_{i} - \beta_{j} \right)^{2} \\ & = {\beta}'(\boldsymbol{I}-\boldsymbol{S}){\beta}\\ & ={\beta}'\boldsymbol{L}{\beta}, \end{aligned}  $$

where *L* is a positive semidefinite matrix derived from the network. The the full model of NET-COX is based on:

12 $$ \begin{aligned} l_{\text{\textsc{NetCox}}}({\beta},h_{0}) = & \sum\limits^{n}_{i=1} \left\{ -\exp(\boldsymbol{X}_{i}'{\beta})H_{0}(t_{i}) + \right. \\ & \left. \qquad \delta_{i} \left[ \log(h_{0}(t_{i}))+\boldsymbol{X}_{i}'{\beta} \right] \right\} \\ & - \frac{1}{2} \lambda {\beta}' \left(\left(1 - \alpha \right) \boldsymbol{L} + \alpha \boldsymbol{I} \right) {\beta}, \end{aligned}  $$

where *λ* is a parameter that controls the penalizing weight of the regularizer and *α* is the parameter that weights the two penalizations.

#### DEGREECOX regression

The function proposed in DEGREECOX combines the total log-likelihood of Cox regression with degree regularization. As previously, the total log-likelihood is calcuted using the Breslow estimator (Eq. 8). The novelty is the introduction of a penalizing term that conveys a vertex centrality information of the subjacent network. To this purpose, both Gene Co-expression Networks (GCN) and Gene Functional Maps (GFM) are used in order to extract the corresponding vertex centrality information. More specifically, each of the different network centrality measures is tested for each of the two networks.

More formally, we introduce a network degree-based constraint to the Cox model that uses the function *Υ*(*β*) as additional cost function:

13 $$  \Upsilon({\beta}) = \sum\limits^{p}_{i=1} {\beta_{i}^{2}} d_{ii} = \beta'\boldsymbol{D}\beta.  $$

where ***D*** is a diagonal matrix with $D_{ii}^{-1}= \sum ^{p}_{j=1}s_{ij}$, i.e., the inverse of the vertex weighted degree.

**Fig. 2:**
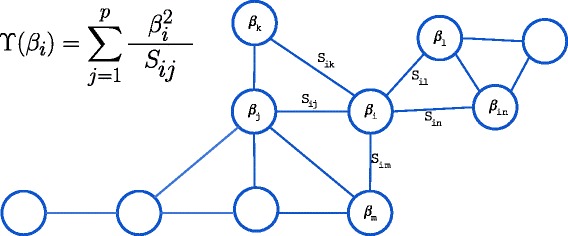
DEGREECOX network regularizer

Figure 2 illustrates this measure, that will be used in the DEGREECOX method.

When adding the constraint to the Cox model, we get the full likelihood as follows:

14 $$  \begin{aligned} l_{DegreeCox}({\beta},h_{0}) = & \sum\limits^{n}_{i=1} \left\{ -\exp(\boldsymbol{X}_{i}'{\beta})H_{0}(t_{i}) + \right. \\ & \left. \qquad \delta_{i}\ \left [ \log(h_{0}(t_{i}))+\boldsymbol{X}_{i}'{\beta} \right ] \right\} \\ & - \frac{1}{2}\lambda\left ({\beta'\boldsymbol{D}\beta} \right). \end{aligned}  $$

This model adds a cost for each gene/vertex that increases as its coefficient *β*_*i*_ increases, but is also inversely proportional to how well connected that vertex is in the graph, given by its degree. Thus, the objective function drives the assignment of larger coefficients to genes that are highly connected in the network. The rationale behind the application of this regularizer is then to identify a set of genes that not only predicts the survival, but that also has a relevant role in the underlying network.

## Results and discussion

In the following experiments, the DEGREECOX, NET-COX, LASSO and RIDGE models were applied to ovarian cancer gene expression datasets. The experiments ran with multiple parameter values, which were selected using the same cross-validation technique as described in [14]. The selected models were then evaluated by comparing the prognostic risk of each patient in the sample, the obtained clustering in high and low risk groups based on Kaplan-Meier estimators [34] and log-rank tests. Analysis of the deviance residues [35] and the concordance c-index of the selected models [36] is also presented for all combinations of datasets and methods.

### Datasets and networks

The three datasets used in these experiments, hereafter named *Bonome*, *TCGA* and *Tothill*, are publicly available from three independent ovarian cancer studies [20–22]. All three contain gene expression data and survival follow-up times for each patient in the study. The datasets were obtained from the HG-U133A platform and the raw files were normalized using the Robust Multichip Average (RMA) preprocessing methodology.

The *Bonome* dataset comprises the follow-up time, survival status and microarray gene expressions for 185 patients. The microarray data contain 12,442 gene expression levels [21]. The *TCGA* dataset comprises the follow-up time, survival status and microarray gene expression of 517 patients and the microarray data contain 12,042 gene expression levels [20]. The *Tothill* dataset also comprises the follow-up time, survival status and microarray gene expression of 278 patients and 19,816 gene expression levels [22]. These three datasets have 6,965 genes in common that were therefore adopted for all the experiments using the Gene Co-expression Network. The same number of genes are present in the Gene Functional Network, which will be considered the benchmark to determine and confirm the weighted degree as the best centrality measure to be used in DEGREECOX.

High edge weights imply a strong connection between the corresponding genes/vertices. This is desirable for centrality measures such as the weighted degree. However, for the betweenness and closeness centrality measures, this would lead to more highly connected vertices having lower betweenness, since they will not be present in the shortest paths. In order to include these strongly connected vertices, the following transformation is applied in these cases:

15 $$  s_{ij}' = \log \left(\frac{1}{s_{ij}} \right).  $$

### Centrality measures evaluation

In order to choose the most adequate centrality measure for the regularization, several tests where performed regarding the topological and connectivity properties of each network. The Gene Co-expression Networks and Gene Functional Networks have an edge between any pair of genes and, as a consequence, the diameter of the networks is 1, making the centrality metrics based on shortest paths or unweighted degree uninformative. In order to tackle this problem, the original networks were split into sub-networks by ranking the edges on their weight and removing them if *s*_*ij*_ was below a given threshold. By working with both the full network and smaller sub-networks, we can attempt to better understand their structure.

The full network had 28,588,141 edges and was progressively reduced using this method, by applying a threshold that varied between 0 (full network) and 1 (fully disconnected). Each sub-network was then studied in terms of its diameter, power law distribution and, for a ranking of the vertices, according to their degree, weighted degree, betweenness and closeness centrality measures.

**Fig. 3:**
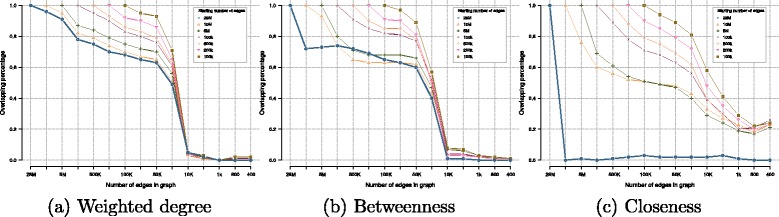
Comparison of the fraction of top-ranked genes calculated for starting networks for the centrality metrics analized: **a** weighted degree, **b** betweenness and **c** closeness. Sub-network properties obtained by removing edges from the starting network

In Fig. 3, we show how varying this threshold affects the top ranking genes for the centrality measures described and the total number of edges kept.

Two criteria for selecting the best centrality measure are evaluated: 1) observing which metric better overlaps the top ranking genes across metrics can help identify a good candidate to test the proposed regularization method; and 2) looking into how rankings change for each metric as the number of edges is reduced should also give insight into the best candidate.

For the first criterion, we take the 1,000 top-ranking genes over the studied metrics and analyse their overlap. While the weighted degree and closeness have 90 *%* of common genes, the betweenness overlaps with less than 45 *%* of either the closeness or weighted degree. We can assess that the weighted degree and closeness hold similar information as they value vertices that are well connected in the network, locally for the first one and globally for the latter. It is interesting how a local measure such as the degree of a vertex gives similar results as when using a global measure as is the closeness.

The second criterion is studied in Fig. 3, which denotes the percentage of top-ranking genes that are kept with different measures as edges are being removed. A ranking of the top 200 genes was calculated for all sub-networks (represented in the x-axis). Each line denotes a different starting network and shows the fraction of the top-ranked genes that are kept as edges are removed. The data shown in Fig. 3 indicate that the betweenness centrality does not perform well with the full graph or big sub-networks as the overlap deteriorates quite fast. On the other hand, weighted degree and closeness show that the top-ranking genes are mostly kept while removing edges, until reaching a critical point near the sub-network with 1,000 edges.

Combining all information, we decided to choose weighted degree as the network-based regularizer to be used (DEGREECOX). It combines local and global information on the network due to its similarity with the closeness measure. The degree is more robust and predictable on the impact of edge removal as well as it is cheaper to compute.

### Performance evaluation of the Cox models

With the best candidate metric selected, experiments were carried out with DEGREECOX using the weighted degree of the network and compared against three existing models: NET-COX, LASSO and RIDGE. The latter two are sub-cases of the elastic net with regularization parameters *α*=1 and *α*=0, respectively. The other parameters for the models were selected using five-fold cross-validation, following the same procedure previously used [14].

In the cross-validation procedure, the dataset is partitioned in 5 different folds, where four of them are used in model training to find the model’s coefficients ($\hat {{\beta }}_{\lambda \alpha }^{(-i)}$) and the *i*-th set is left out. This procedure is performed 5 times for each (*λ*,*α*) parameter combination, or (*λ*), depending on the model. The test itself will determine the parameters that best fit the training data and perform best to new unseen data. This is done by maximizing the partial likelihood (*pl*) between the full dataset (**X**) and the *pl* of the test set (**X**_(−*i*)_).

16 $$ CV(\lambda,\alpha) = \sum_{i = 1}^{5} \left [ pl \left (\mathbf{X},\hat{{\beta}}_{\lambda\alpha}^{(-i)} \right) - pl \left (\mathbf{X_{(-i)}},\hat{{\beta}}_{\lambda\alpha}^{(-i)} \right) \right ].  $$

Three different analytical methods were used to evaluate the models: the root mean squared error (*RMSE*); the concordance index (c-index); and the Kaplan-Meier estimator.

The residuals used to calculate the RMSE were the deviance residuals [37], that calculate the difference between the log-likelihood (Eq. 6) for each individual in the dataset using the global inferred model ($\hat {\beta }$) and a *saturated*, or full model, ($\dot {\beta }$). The *saturated* model is a perfect fit for the data, as the *β* coefficients are allowed to be different for each individual. This residual is centered in zero and can be regarded as the generalization of the residual sum of squares [37]:

17 $$  res_{Deviance} = -2 \log(l(\hat{\beta})) - \log(l(\dot{\beta})).  $$

The concordance c-index [38] is a relative measure that will assess all permissible pairs of individuals in the sample and compare if their survival time is in line with the hazard relative risk. Pairs where both individuals are censored or when only one is censored and has a shorter time than the uncensored are not considered valid. The algorithm increases a concordance count by 1 with every pair that is in one of three cases: (a) individual with higher risk has shorter survival time; (b) hazard risks and survival time are the same; (c) one individual is censored and has a lower risk. Otherwise the count is increased by 0.5. The c-index is calculated by dividing the count by the number of permissible pairs [38].

The Kaplan-Meier estimator [34] is a non-parametric method that estimates the survival function, providing information, at any time point in the data, about the fraction of individuals where the event did not occur. It allows for right censored data and, when calculated for two different groups, we use the log-rank test [39] to compare survival distributions.

In order to test the predictability of the models the following procedure was used: find the best parameters for a training dataset using 5-fold cross validation and then test on the same dataset and 2 others. For example train a model with *Bonome* to test with the *TCGA* and *Tothill* dataset.

The results obtained are summarized in Tables 1 and 2 to assess the generalization capability of the methods with new data and how it fits with the training set.

**Table 1:** Deviance and C-index results for models chosen by 5-fold cross-validation and tested on all 3 datasets (including 2 that were hidden from the training phase). The LASSO and RIDGE methods do not use network information so the values for GCN and GFM are the same, they are only shown in both networks when they are better than DEGREECOX and NET-COX

	*Train*	Bonome						TCGA						Tothill					
	*Test*	Bonome		TCGA		Tothill		Bonome		TCGA		Tothill		Bonome		TCGA		Tothill	
	*Network*	GCN	GFM	GCN	GFM	GCN	GFM	GCN	GFM	GCN	GFM	GCN	GFM	GCN	GFM	GCN	GFM	GCN	GFM
RMSE	DegreeCox	**0** **.** **5** **5** **8** **1**	**0** **.** **7** **7** **2** **4**	1.3189	1.1538	1.2139	1.1027	**1** **.** **2** **3** **6** **7**	1.3619	0.9201	0.8043	**1** **.** **0** **5** **7** **3**	1.1083	1.6326	1.2975	1.3749	1.1679	**0** **.** **5** **1** **1** **6**	0.7013
	Net-Cox	0.8131	0.8353	1.1438	**1** **.** **1** **2** **8** **5**	1.0992	**1** **.** **0** **8** **8** **6**	1.3514	**1** **.** **3** **0** **4** **5**	0.8361	0.8508	1.1003	**1** **.** **0** **8** **0** **2**	**1** **.** **2** **9** **1** **7**	**1** **.** **2** **5** **9** **1**	**1** **.** **1** **6** **1** **2**	**1** **.** **1** **4** **0** **3**	0.7363	0.7606
	Ridge	0.7807		**1** **.** **1** **4** **1** **3**		**1** **.** **0** **9** **8** **6**		1.3755		**0** **.** **7** **2** **1** **5**		1.1769		1.5649		1.3252			**0** **.** **5** **4** **3** **2**
	Lasso	0.7887		1.4619		1.2586		1.7419		0.8105		1.3019		1.9595		1.4208		0.5444	
C-Index	DegreeCox	**0** **.** **9** **7** **9** **5**	0.9401	0.6020	0.6037	0.6455	0.6494	0.6444	0.6427	0.8476	0.9089	**0** **.** **6** **7** **1** **1**	0.6695	0.6011	0.6088	0.6100	0.6215	**0** **.** **9** **8** **3** **4**	0.9519
	Net-Cox	0.9260	0.9202	0.6079	0.6054	0.6483	0.6506	0.6416	0.6439	0.8918	0.8892	0.6633	**0** **.** **6** **7** **0** **5**	**0** **.** **6** **1** **5** **2**	**0** **.** **6** **1** **0** **6**	**0** **.** **6** **2** **4** **4**	**0** **.** **6** **2** **5** **0**	0.9389	0.9352
	Ridge		**0** **.** **9** **4** **1** **0**	**0** **.** **6** **1** **7** **7**		**0** **.** **6** **5** **6** **9**		**0** **.** **6** **4** **9** **2**		**0** **.** **9** **3** **9** **4**		0.6579		0.6000		0.5926			**0** **.** **9** **8** **2** **9**
	Lasso	0.9309		0.5615		0.6124		0.6405		0.9043		0.6399		0.5075		0.5728		0.9784	

Values in bold represent the best performing method for the dataset/network combination (per RMSE and C-Index)

**Table 2:** *P*-values for log-rank test results for models chosen by 5-fold cross-validation and tested on all 3 datasets (including 2 that were hidden from the training phase). The log-rank tests the separation in two categories of patients, high and low risk based on the expression dataset, using the top and lower 40 *%* PI groups and the top and lower 50 *%* PI groups. The LASSO and RIDGE methods do not use network information so the values for GCN and GFM are the same, they are only shown in both networks when they are better than DEGREECOX and NET-COX. The *p*-values when the model is tested on the same dataset used in training are always 0 and are ommited from the table

	*Train*	Bonome				TCGA				Tothill			
	*Test*	TCGA		Tothill		Bonome		Tothill		Bonome		TCGA	
	*Network*	GCN	GFM	GCN	GFM	GCN	GFM	GCN	GFM	GCN	GFM	GCN	GFM
40 *%*PI Thres.	DegreeCox	2.084*E*-5	2.124*E*-5	0.0013	4.390*E*-4	2.046*E*-4	3.990*E*-4	**6** **.** **5** **4** **7** **E** **−** **6**	5.822*E*-6	**9** **.** **8** **3** **3** **E**-**5**	**1** **.** **7** **5** **7** **E**-**4**	8.347*E*-8	3.125*E*-8
	Net-Cox	1.082*E*-4	2.791*E*-5	7.726*E*-4	**1** **.** **5** **1** **4** **E**-**4**	2.815*E*-4	1.185*E*-4	4.241*E*-5	**1** **.** **4** **3** **2** **E**-**6**	1.696*E*-4	2.545*E*-4	**7** **.** **7** **1** **7** **E**-**9**	**4** **.** **5** **0** **3** **E**-**9**
	Ridge	**1** **.** **5** **9** **4** **E**-**6**		**2** **.** **5** **3** **7** **E**-**4**		4.233*E*-4		1.765*E*-5		0.0016		1.864*E*-5	
	Lasso	0.0364		0.0048		**7** **.** **4** **3** **6** **E**-**5**		0.0036		0.5630		0.0033	
50 *%*PI Thres.	DegreeCox	3.332*E*-4	5.284*E*-5	0.0076	0.0084	4.394*E*-4	0.0090	**5** **.** **7** **8** **1** **E**-**5**	**1** **.** **3** **0** **9** **E**-**4**	0.0045	**4** **.** **3** **0** **2** **E**-**4**	5.264*E*-7	7.183*E*-7
	Net-Cox	2.169*E*-5	5.086*E*-5	0.0170	0.0179	0.0036	0.0015	1.247*E*-4	3.126*E*-4	**0** **.** **0** **0** **2** **6**	8.138*E*-4	**1** **.** **1** **0** **5** **E**-**8**	**1** **.** **6** **3** **2** **E**-**7**
	Ridge	**1** **.** **7** **9** **5** **E**-**5**		**0** **.** **0** **0** **1** **3**		**3** **.** **1** **9** **3** **E**-**4**		0.0029		0.0050		3.499*E*-5	
	Lasso	0.0720		0.0048		0.0022		0.0193		0.6464		0.0050	

Values in bold represent the best method for the dataset/network combination (per 40 % and 50 % separation)

We observe that DEGREECOX, NET-COX and RIDGE regression perform very similarly across all three evaluation measurements. Regarding the deviances, as measured by RSME, we can conclude that network information improves the results in all the datasets except for *TCGA* tested on *TCGA*, where RIDGE achieves lower deviances. For the *Bonome* and *Tothill* datasets, DEGREECOX has the best results. When using cross-testing, NET-COX has the best results for the *Bonome* and *Tothill* datasets and DEGREECOX for the *TCGA* dataset. NET-COX determines a very good model using the *Tothill* dataset as training, but then alternates with RIDGE and DEGREECOX on the other datasets. Such similar results are expected, as both NET-COX and DEGREECOX use the same additional information, namely the GCN and GFM networks. The small difference in the results could be explained by how the networks are being used. While NET-COX takes the weighted edges of every two genes, DEGREECOX takes the sum for every vertex losing some detail in the process. However, this does not seem relevant as the difference in the deviance is not significant.

**Fig. 4:**
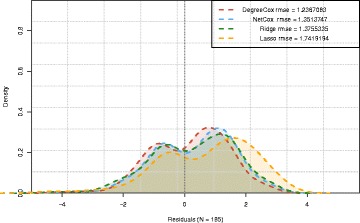
Residuals when models are trained with the correlation network and *TCGA* dataset and tested with the *Bonome* dataset

To further evaluate how these accuracy measures vary, we assessed the distribution of the residuals for the different methods. In Fig. 4, we show a typical result obtained when applying the four studied models on the *TCGA*/*Bonome* example. This illustrates that all the residuals exhibit a bimodal distribution. However DEGREECOX leads to a smaller variance and LASSO presents the highest dispersion of RMSE values.

The results are slightly different when observing the concordance c-index. The results of RIDGE are consistently better than those of both NET-COX and DEGREECOX. Although the difference is small, at most of 2 *%*, between the models. LASSO continues to perform worse than the other models with this evaluation measure.

Finally, the comparison between the methods involved the evaluation of their potential to correctly classify patients accordingly to their survival risks. This was performed by dividing the samples into two groups, high and low risk individuals, based on each individuals’ estimated hazard function and using a given (optimal) threshold. This value, called prognostic index (*PI*), is estimated, for each model, by choosing the threshold for $PI_{n} = \sum ^{P}_{i = 1}X_{{i,n}} \cdot \beta _{i}$ that leads to the lowest *p*-value, as assessed by the log-rank test.

**Fig. 5:**
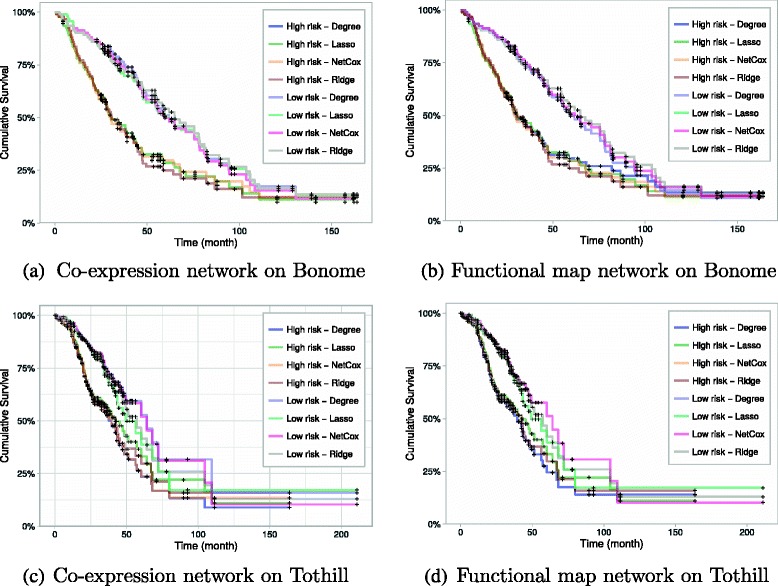
Kaplan-Meier curves for high vs. low risk groups with the model learnt from the *TCGA* dataset and tested on *Bonome* (**a** and **b**) and *Tothill* (**c** and **d**). When a death event occurs for an individual, the cumulative survival decreases

We stratified the patients as in the NET-COX proposal (Zhang et al. 2013), by assigning those with the lowest 40 *%* PI to the low-risk group, and the top 40 *%* PI to the high-risk group. The results obtained by using a 50 *%*- 50 *%* stratification are also reported since they correspond to a less favorable partition of the patients, by including those with intermediate risks. Then, the Kaplan-Meier curves are estimated (Fig. 5) and log-rank tests performed, all available as Additional file 1.

The analysis was done for each model and shows that when testing with the *Bonome* and *TCGA* datasets, there is a statistically significant difference between the survival functions of the two groups across all models. The dataset that had the worst separation was the *Tothill* one, as LASSO and RIDGE perform in a similar way to the other methods up until month 30, which can be seen in Fig. 5 c and d. Afterwards, both curves start to converge to each other. This observation is coherent with the *p*-value results of the log-rank test in Table 2. This result in particular shows that enriching the models with network-based information can lead to better predictive models.

When measuring the separation between two groups by assessing the *p*-value of the log-rank test, there is a slight improvement in the results of DEGREECOX for the 50 *%*- 50 *%* partition over the top 40 *%*-lower 40 *%* case (where 20 *%* of the observations are excluded), which might indicate a better performance in the presence of noisy information. This will be further explored in the future. For the 50 *%*- 50 *%* partition and considering the log-rank tests, RIDGE regression achieves the lowest *p*-values in half of the tests. Comparing the methods that use network information in this experimental setting, DEGREECOX achieves better results than NET-COX for the majority of the combinations (except for *Tothill* training and testing on in the *TCGA*).

The separation of high and low risk patients is statistically significant although it could be improved by adding as variables to the model physiological characteristics, such as tumour stage, age groups, ethnicities or gender. These are not currently included, as proposing a new classifier is out of the scope of the present work, that instead, introduces a new regularization model that requires further research.

The results obtained in this study for the NET-COX model are comparable with those of the original paper [14] using all the genes (see the Additional file 1). The obtained *p*-value results are of the same order of magnitude between both experiments, with the small differences being explained by differences in the pre-processing.

Although none of the methods seems to perform better in all situations, we can conclude that including network information does not deteriorate the accuracy and can provide better interpretability of the obtained Cox survival models, which will be further explored in future work.

## Conclusions

We proposed DEGREECOX, a novel method to estimate survival models using network-based regularization. The results show that DEGREECOX consistently performs as well as NET-COX and RIDGE in all scenarios and with better results against LASSO. The evaluation was performed using deviance residuals and the log-rank test of the Kaplan-Meier estimator for two different groups, high risk and low risk individuals, and this is somewhat expected as all three methods are based on the same norm.

These methods show promising results, and possible extensions can include more topological and network measures. Other models beyond Cox can also be easily integrated in this framework. The analysis of different types of network properties can also be tested further, and combining different regularizers may lead to an improvement of the classification accuracy.

## Additional file

Additional file 1 Kaplan Meier curves and log-rank tests. A PDF file that includes figures of Kaplan-Meier curves and log-rank tests obtained for all the combinations of the three datasets (Bonome, TCGA, Tothill) that are described in the manuscript. (PDF 2682 kb)
