# Supplementary material for: DegreeCox – a network-based regularization method for survival analysis
Source: BMC Bioinformatics. 2016 Dec 13;17(Suppl 16):449. doi: 10.1186/s12859-016-1310-4 (PMC5249012; doi:10.1186/s12859-016-1310-4)

SUPPLEMENTARY MATERIAL

DegreeCox: a network-based regularization method for survival analysis

André Veríssimo, Arlindo L. Oliveira, Marie-France Sagot and Susana Vinga

Kaplan Meier curves and log-rank tests

The following figures are the Kaplan-Meier curves and log-rank tests obtained for all the combinations of the three datasets (Bonome, TCGA, Tothill) described in the manuscript. The testing was performed using a each of the datasets for training and testing the results on the other two. Two stratifications were performed: A) Using the top 40% patients with higher risk *versus* the 40% low risk patients; and B) Using a 50%-50% split.

A) Top 40% *vs.* Lower 40%

A1) Training dataset: Bonome

Testing dataset: TCGA

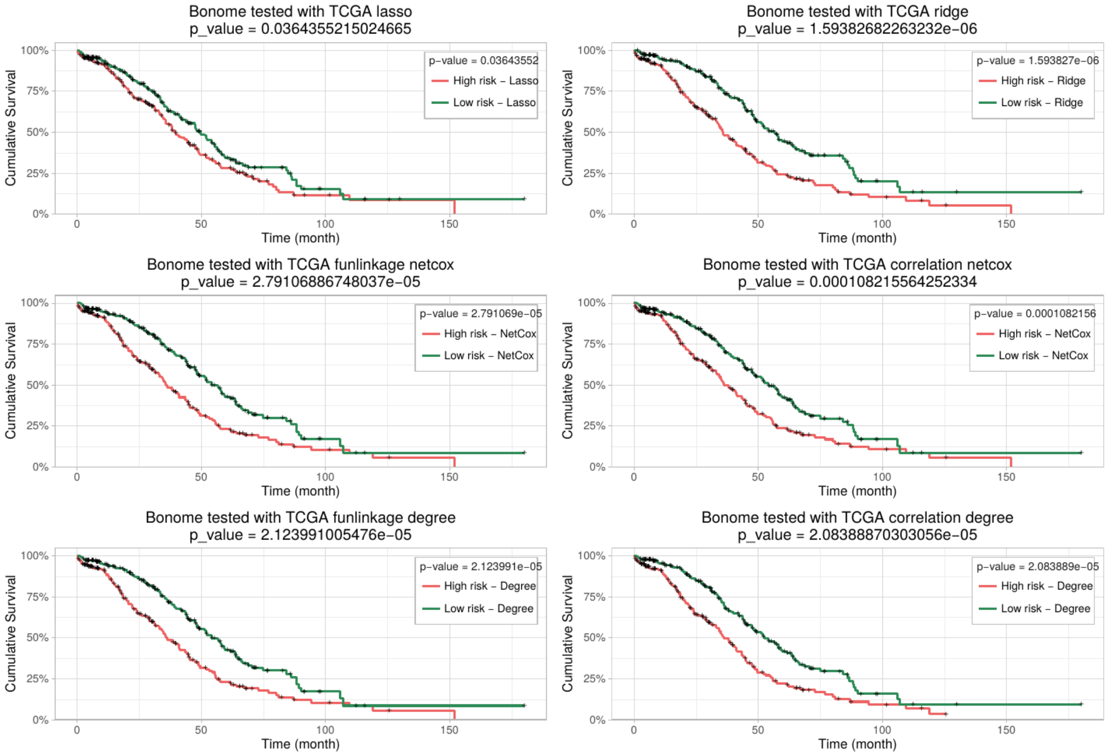

Testing dataset: Tothill

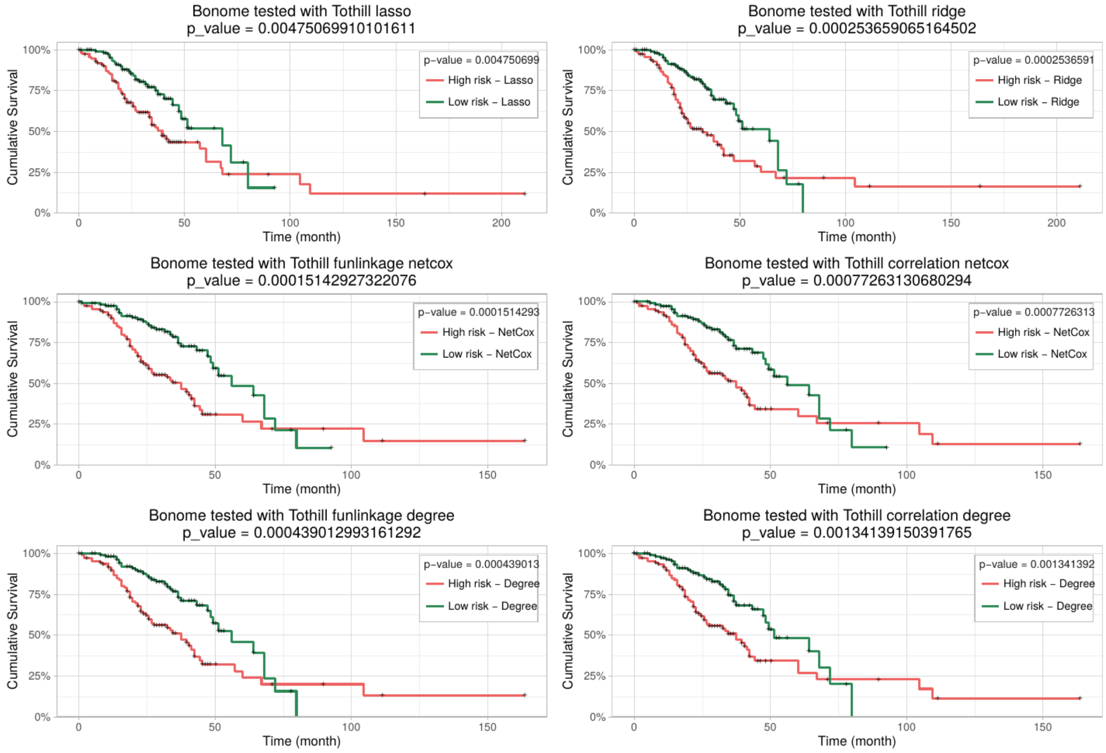

A2) Training dataset: TCGA

Testing dataset: Bonome

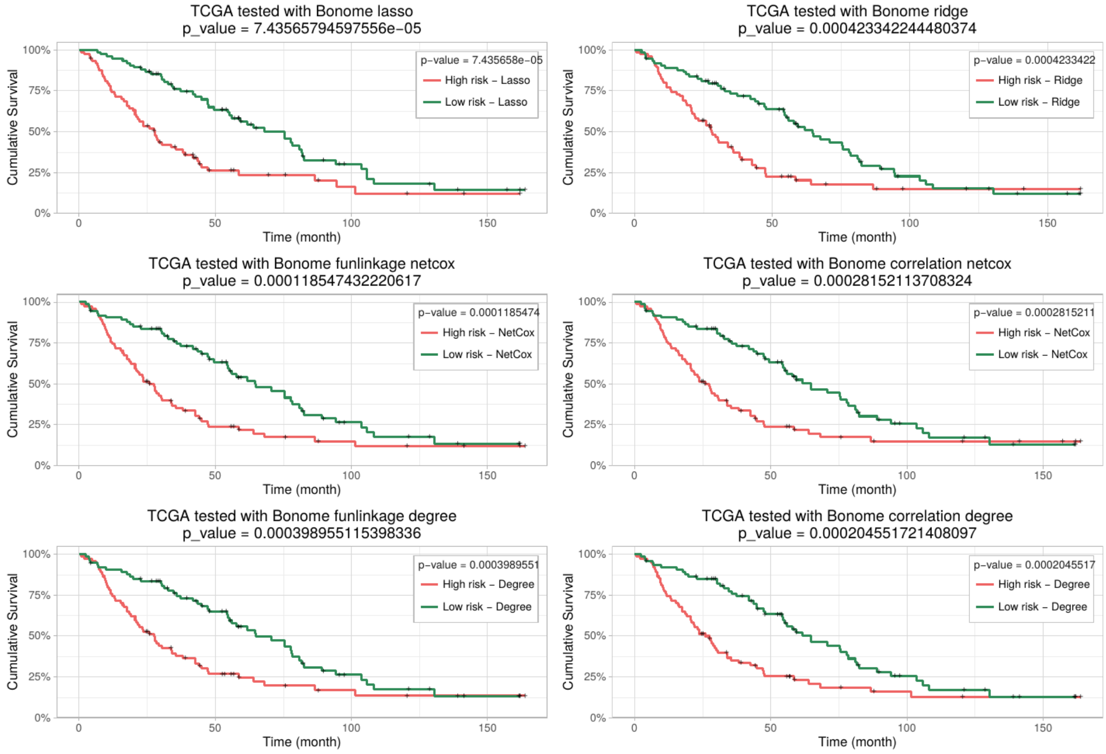

Testing dataset: Tothill

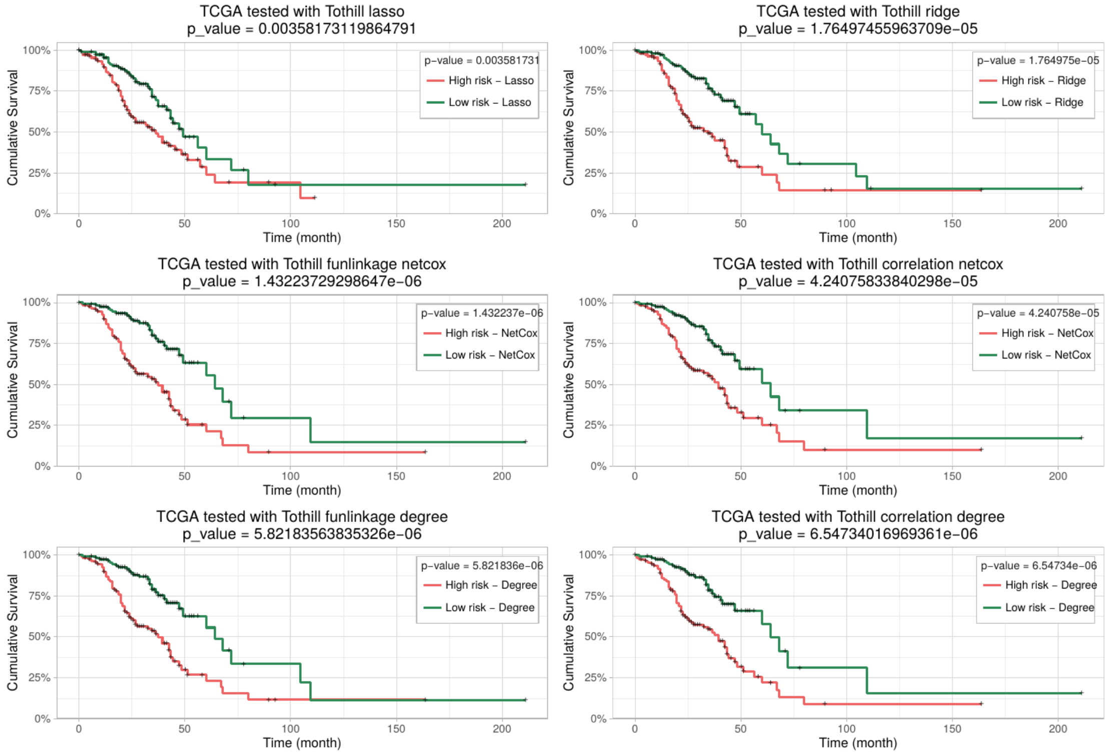

A3) Training dataset: Tothill

Testing dataset: Bonome

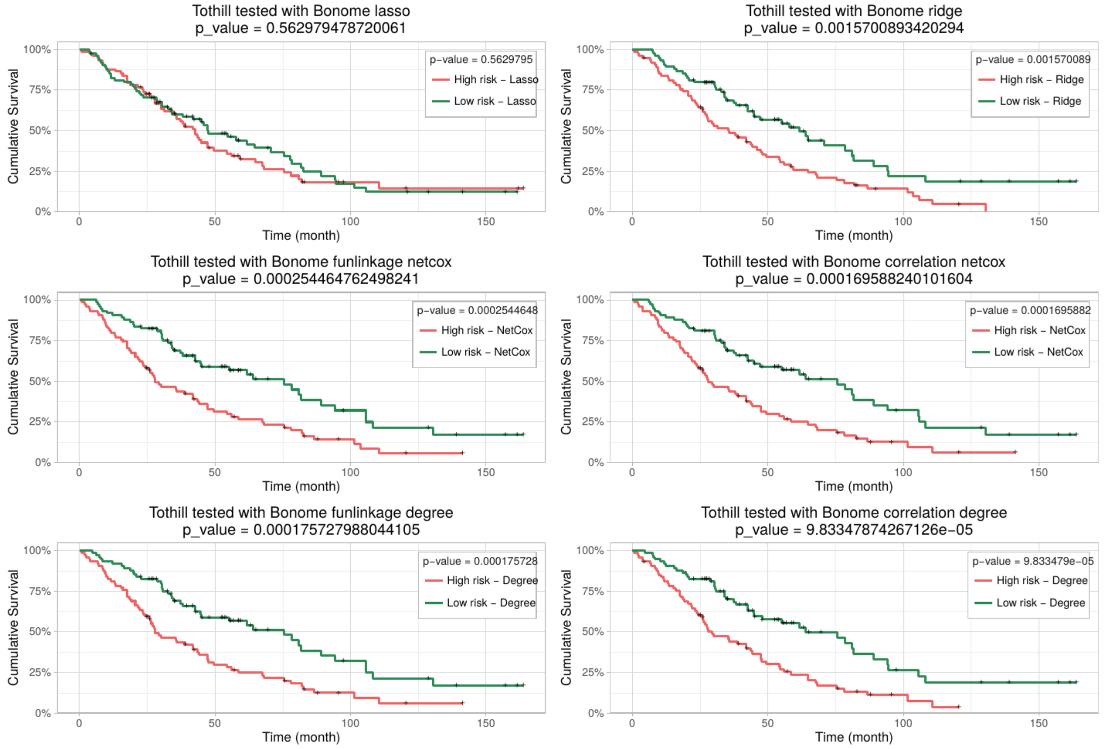

Testing dataset: TCGA

Tothill tested with TCGA lasso
p_value = 0.00333422770723957

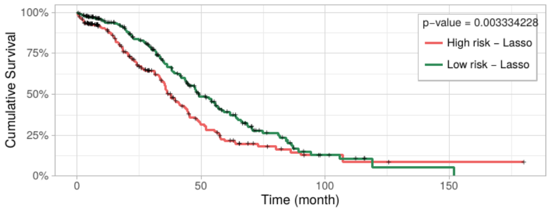

Tothill tested with TCGA ridge
p_value = 1.86381501240396e−05

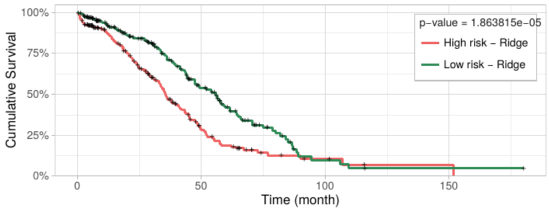

Tothill tested with TCGA funlinkage netcox
p_value = 4.50323323075708e−09

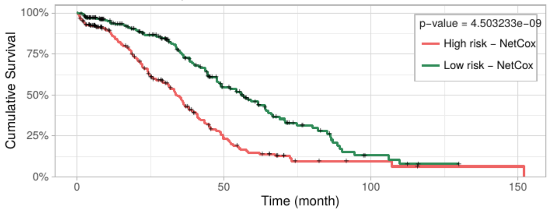

Tothill tested with TCGA correlation netcox
p_value = 7.71737296290098e−09

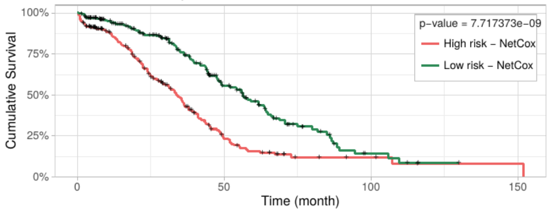

Tothill tested with TCGA funlinkage degree
p_value = 3.12454048190247e−08

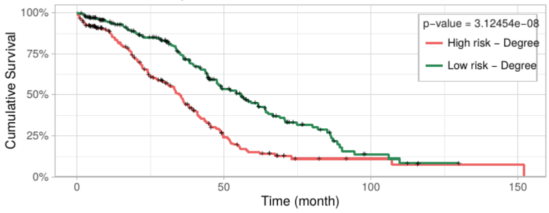

Tothill tested with TCGA correlation degree
p_value = 8.3474358869573e−08

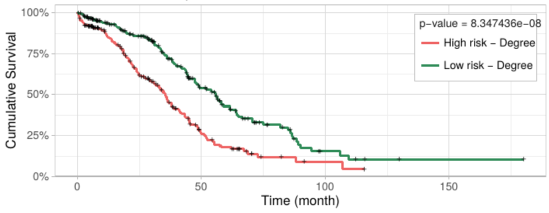

B) Top 50% *vs.* Lower 50%

B1) Training dataset: Bonome

Testing dataset: TCGA

Bonome tested with TCGA lasso
p_value = 0.0719936836080244

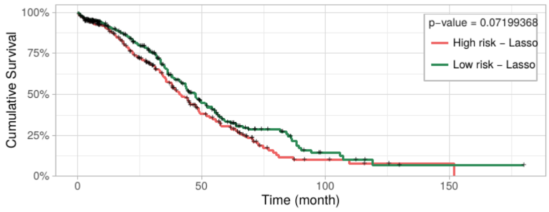

Bonome tested with TCGA ridge
p_value = 1.7952804948429e−05

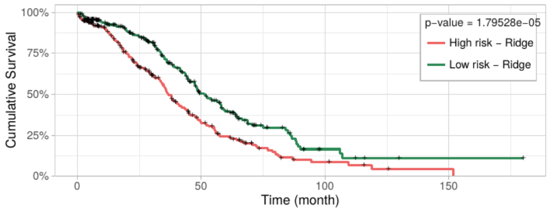

Bonome tested with TCGA funlinkage netcox
p_value = 5.08649318814447e−05

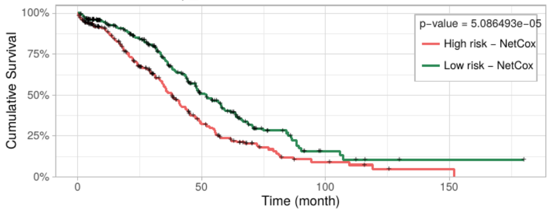

Bonome tested with TCGA correlation netcox
p_value = 2.16929606665639e−05

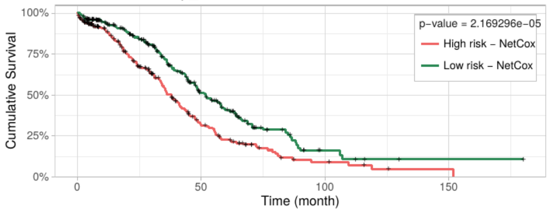

Bonome tested with TCGA funlinkage degree
p_value = 5.28367840382238e−05

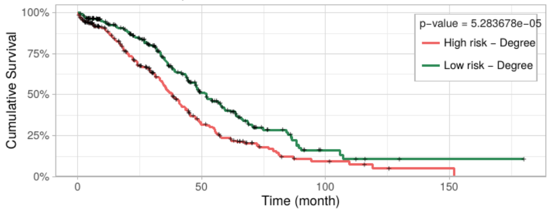

Bonome tested with TCGA correlation degree
p_value = 0.000333220164252412

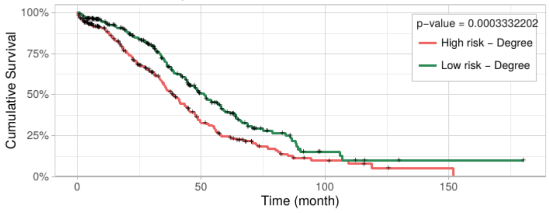

Testing dataset: Tothill

Bonome tested with Tothill lasso
p_value = 0.00477812233222352

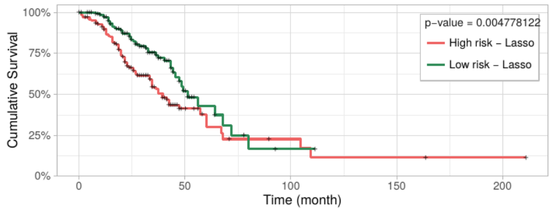

Bonome tested with Tothill ridge
p_value = 0.00124797857886416

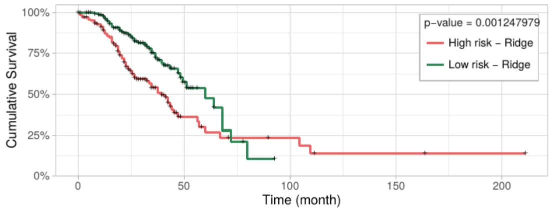

Bonome tested with Tothill funlinkage netcox
p_value = 0.0179248387080944

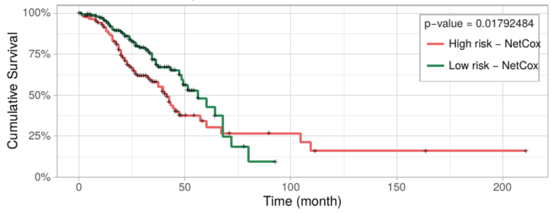

Bonome tested with Tothill correlation netcox
p_value = 0.0170350263698924

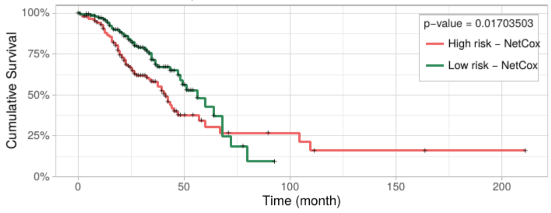

Bonome tested with Tothill funlinkage degree
p_value = 0.00844195932603076

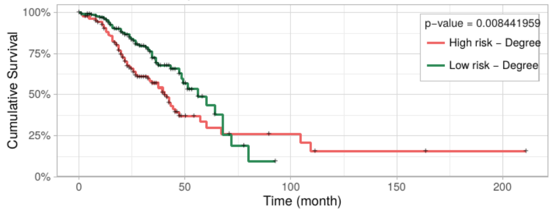

Bonome tested with Tothill correlation degree
p_value = 0.00759515431264868

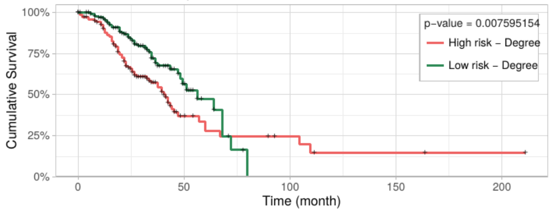

B2) Training dataset: TCGA

Testing dataset: Bonome

TCGA tested with Bonome lasso
p_value = 0.00219540966387699

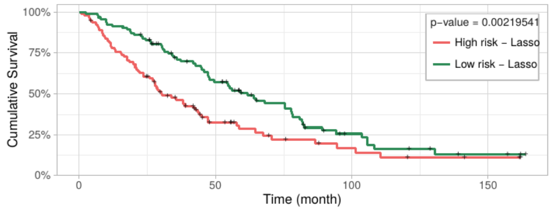

TCGA tested with Bonome ridge
p_value = 0.000319305416062251

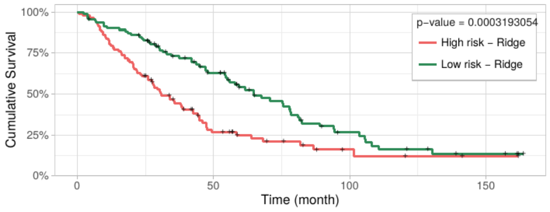

TCGA tested with Bonome funlinkage netcox
p_value = 0.00150351131150595

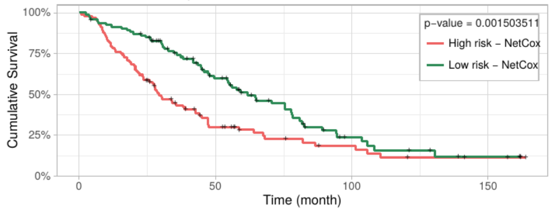

TCGA tested with Bonome correlation netcox
p_value = 0.00362651183838825

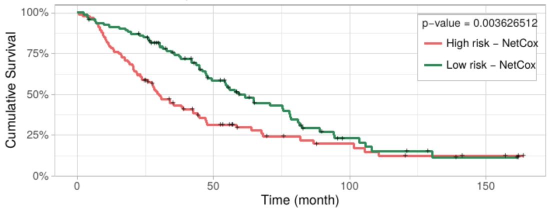

TCGA tested with Bonome funlinkage degree
p_value = 0.00900786861498459

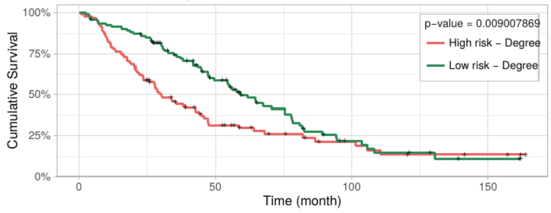

TCGA tested with Bonome correlation degree
p_value = 0.0004393788551319

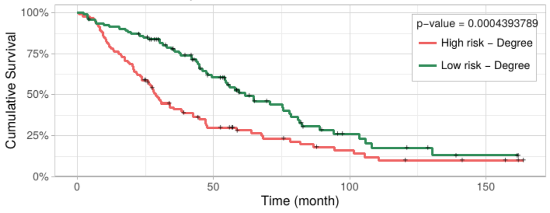

Testing dataset: Tothill

TCGA tested with Tothill lasso
p_value = 0.0193095048158416

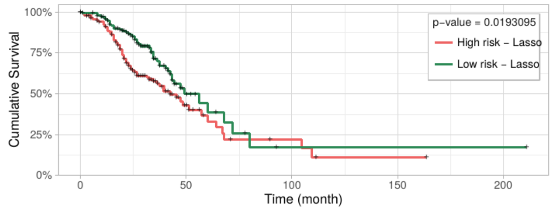

TCGA tested with Tothill ridge
p_value = 0.00285640011936295

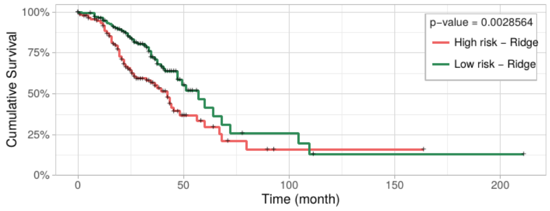

TCGA tested with Tothill funlinkage netcox
p_value = 0.000312605934409294

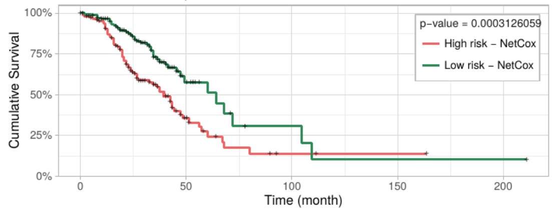

TCGA tested with Tothill correlation netcox
p_value = 0.000124741329649458

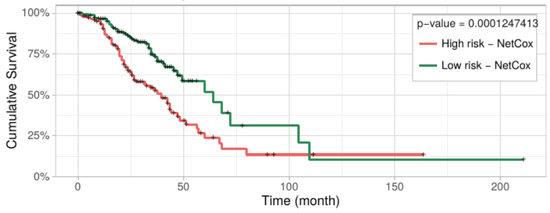

TCGA tested with Tothill funlinkage degree
p_value = 0.000130920888348984

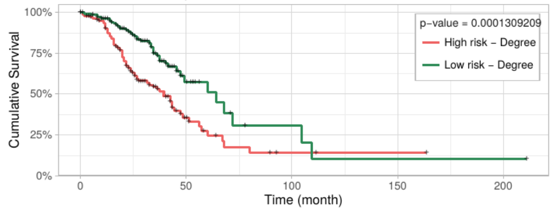

TCGA tested with Tothill correlation degree
p_value = 5.78139813873779e−05

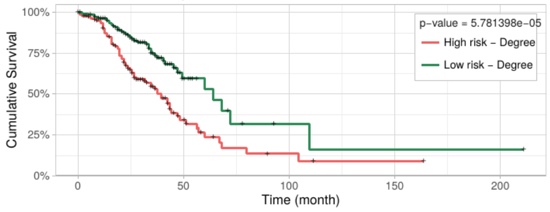

B3) Training dataset: Tothill

Testing dataset: Bonome

Tothill tested with Bonome lasso
p_value = 0.646378766155758

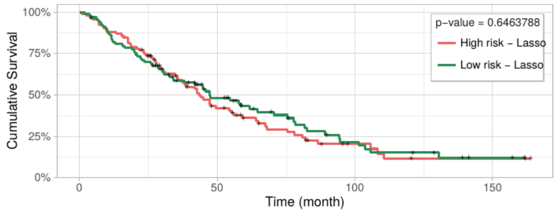

Tothill tested with Bonome ridge
p_value = 0.00500067936240978

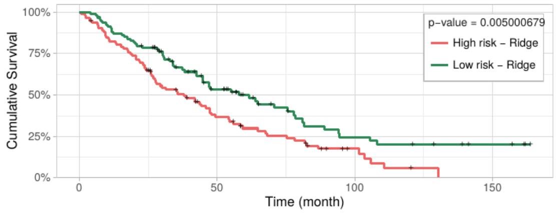

Tothill tested with Bonome funlinkage netcox
p_value = 0.000813766677556949

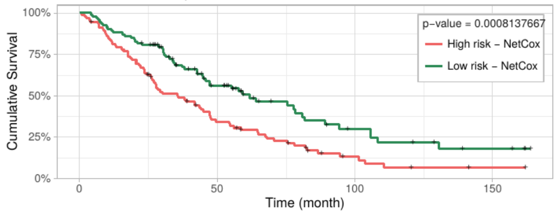

Tothill tested with Bonome correlation netcox
p_value = 0.0026411212371904

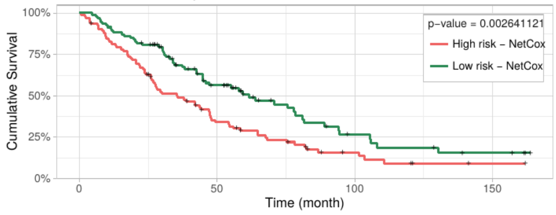

Tothill tested with Bonome funlinkage degree
p_value = 0.000430243359809568

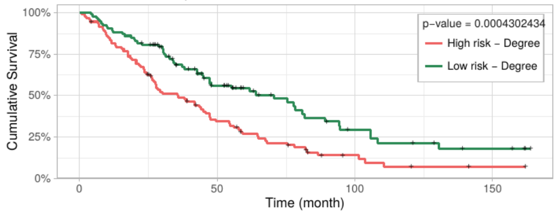

Tothill tested with Bonome correlation degree
p_value = 0.00450557885266367

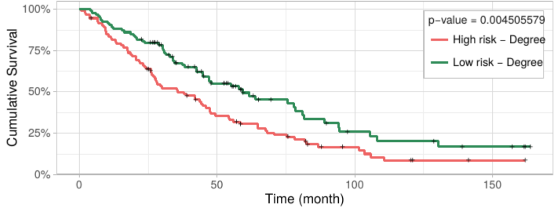

Testing dataset: TCGA

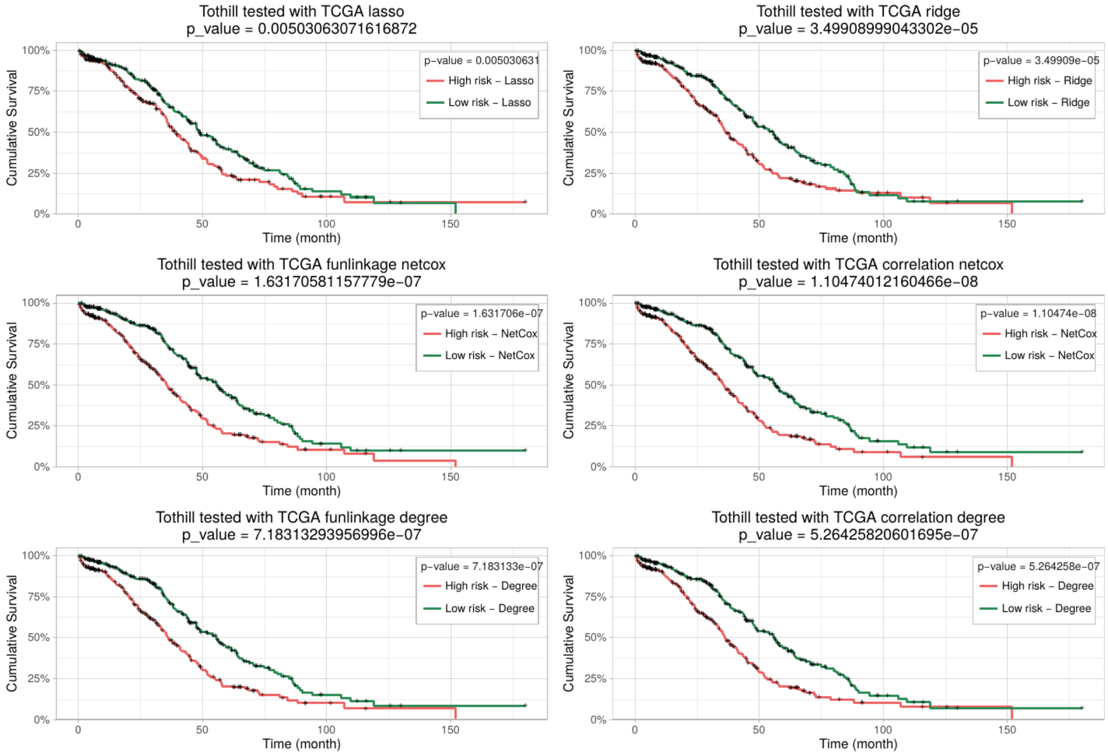

Supplement: Additional file 1 — Kaplan Meier curves and log-rank tests. A PDF file that includes figures of Kaplan-Meier curves and log-rank tests obtained for all the combinations of the three datasets (Bonome, TCGA, Tothill) that are described in the manuscript. (PDF 2682 kb) [file 12859_2016_1310_MOESM1_ESM.pdf]
